# Sensitive Colorimetric Detection of Interleukin-6 *via* Lateral Flow Assay Incorporated Silver Amplification Method

**DOI:** 10.3389/fbioe.2021.778269

**Published:** 2021-11-26

**Authors:** Mohammad Rahbar, Yuling Wu, J. Anand Subramony, Guozhen Liu

**Affiliations:** ^1^ Graduate School of Biomedical Engineering, The University of New South Wales, Kensington, NSW, Australia; ^2^ Integrated Bioanalysis, Clinical Pharmacology and Quantitative Pharmacology, BioPharmaceuticals R&D, AstraZeneca, Gaithersburg, MD, United States; ^3^ Biologics Engineering R&D, AstraZeneca, Gaithersburg, MD, United States; ^4^ School of Life and Health Sciences, The Chinese University of Hong Kong, Shenzhen, China

**Keywords:** point-of-care diagnostics, lateral flow immunoassay, interleukin-6, silver enhancement reagent, paper test strips, colorimetric detection

## Abstract

Interleukin-6 (IL-6) is a pro/anti-inflammatory cytokine, the quantitative detection of which has been extensively considered for diagnosis of inflammatory associated diseases. However, there has not yet been a reliable, low-cost, and user-friendly platform developed for point-of-care (POC) detection of IL-6, which will eliminate the conventional costly, time-consuming, and complex assays. In this work, we developed a lateral flow assay for colorimetric detection of IL-6, using anti-IL-6 antibodies conjugated to gold nanoparticles (AuNPs) as the detection probes. Silver amplification technique was incorporated with the newly developed assay in order to enhance the obtained colorimetric signals, allowing sensitive detection of IL-6 in human serum in the desired physiological ranges (i.e., 5–1000 pg/mL). A limit of detection of 5 pg/mL could be achieved for IL-6 detection in serum with the amplification step which was not achievable in the standard assay. The corresponding specificity and reproducibility tests were all preformed to confirm the reliability of this assay for quantitative measurement of IL-6 in a POC manner.

## Introduction

Inflammatory diseases include conditions that are characterized by inflammation. Examples include allergy, asthma, autoimmune diseases, hepatitis, inflammatory bowel disease, preperfusion injury, and transplant rejection. While many of these are life threating, diseases such as Sepsis are one the most common and major causes of mortality observed all around the world. Thus, early and efficient diagnosis of such health disorders are crucial for higher survival rates of patients including the onset of worsening inflammatory conditions using biomarkers or assays. There have been numerous biomarkers reported in the literature for inflammatory signals, the concentration of which in body fluids is correlated with conditions. Cytokines are one of these protein biomarkers which are present in different biological samples such as serum, blood, saliva, and sweat, representing high correlations with inflammation, especially when they are in abnormal levels (Seruga et al., 2008; Kumari et al., 2016; Kany et al., 2019). Cytokine storm is one type of life-threatening systemic inflammatory syndromes resulting in elevated levels of circulating cytokines. The cytokine storm could significantly increase the mortality and morbidity for the current SARS-CoV-2 infection. (Han et al., 2020; Ragab et al., 20202020). Thus, detection and monitoring of these biomarkers can be useful for diagnosis of these diseases. The current laboratory-based testings of cytokines are extremely time-consuming, involves batch processing of samples, and inefficient, thereby delaying the timely diagnosis process, which can consequently lead to higher risks of death in some cases. Therefore, sensor technologies, especially the point-of-care diagnostic tools have gained extensive attention in order to provide more rapid and robust platforms for detection of the corresponding biomarkers (Stenken and Poschenrieder, 2015; Liu et al., 2021a; Liu et al., 2021b).

Interleukin 6 (IL-6) is a pro/anti-inflammatory cytokine, secreted by immune system cells, which has been extensively considered as a biomarker for diagnosis of inflammation related diseases. IL-6 protein consists of 184 amino acids and has molecular weight of 26 kDa. IL-6 is also a significant element in other physiological disfunctions such as activity of cancer cells. In addition, there have been some interesting reports demonstrating a significant association between inflammation and cancer, resulting from high IL-6 levels in the cancer or disease affected environments (Tanaka et al., 2014). In healthy adults the level of IL-6 is normally in the range of 5–25 pg/mL, whereas higher levels (e.g., up to 1,000 pg/mL) of IL-6 in serum or blood can be associated with the abnormal physical conditions including sepsis or cancer. Our study shows IL-6 level increased more than 5 times in a mice model with Parkinson’s disease by comparing with a control mice group. (Shen et al., 2020). It has been reported several times that IL-6 level above 500 pg/mL can be extremely vital, leading to death in many cases. Since IL-6 is present in very low levels (i.e., pg/mL ranges) in extracellular environment, sensitive, rapid, and accurate detection of IL-6 protein can be challenging (Liu et al., 2021a). Another key indication associated with excess cytokines is called cytokine release syndrome (CRS), an acute systemic inflammatory syndrome characterized by fever that could be due to after treatment effects with certain types of immunotherapy, such as monoclonal antibodies and CAR-T cells. IL-6 seems to hold a key role in CRS pathophysiology since highly elevated IL-6 levels are seen in patients with CRS. In essence, cytokine release syndrome is caused by a large, rapid release of cytokines into the blood from immune cells affected by the immunotherapy. Conventional batch process of cytokine assays such as enzyme-linked immunosorbent assay (ELISA) cannot measure CRS and its onset in real time leading to multiple complications for patients. The non-real time measurement also does not allow adaptive therapy to patients on time.

There have been various platforms developed for quantitative detection of IL-6, and ELISA can be regarded as the most conventional and standard technique in this regard (Liu et al., 2017; Qi et al., 2017; Russell et al., 2019). However, the available conventional methods rely on expensive or bulky instruments, trained personnel, complex and time-consuming assays, which make them unsuitable for point-of-care (POC) detection of this biomarker. Therefore, development of a viable platform for POC quantitative determination of IL-6 will be highly advantages for early diagnosis of disease states and real time management of patients.

Lateral flow immunoassays (LFAs), have been very well developed and established for a wide variety of applications including clinical diagnostics, food, and water samples testings, and environmental monitoring. LFAs offers some unique advantages such as simplicity, user-friendliness, low-cost, and easy fabrication process, which make them ideal platforms for POC testings. A standard LFA includes a reporter probe capable of interacting with the target analyte followed by a secondary interaction at the test zone (T-line) on the nitrocellulose membrane, indicating the assay signal (Wang et al., 2016; Huang et al., 2020a; Shirshahi and Liu, 2021). Different types of LFAs were developed for detection of IL-6 (Wang et al., 2019; Huang et al., 2020b; Ruppert et al., 2020). A doubled-antibody based LFAs was developed using europium nanoparticles as the fluorescence signal read-out with a wide linear range (2–500 pg/mL) and a good sensitivity (0.37 pg/mL) (Huang et al., 2020b). Quantum dots were also used as the fluorescence probe in LFA for detection of IL-6 with a sensitivity is 0–20 nmol/L (Ruppert et al., 2020). A surface-enhanced Raman scattering (SERS)-based lateral flow assay (LFA) is developed for the quantitative analysis of IL-6 with a linear range from 1 pg/mL to 1 μg/mL, and the sensitivity of IL-6 in whole blood is 5 pg/mL (Wang et al., 2019). Even though, there have been so many different types of colorimetric, fluorometric or SERS labels used as a reporter in LFAs, gold nanoparticles (AuNPs) have gained high popularity in this regard due to multiple features such as easy preparation, high affinity for the detection antibodies, and the possibility for naked-eye colorimetric detection. However, when it comes to highly sensitive quantitative analysis of some biomarkers in low abundance such as cytokines, AuNPs are not fully capable of covering the desired demand of sensitive detections (Bahadır and Sezgintürk, 2016; Borse et al., 2020). Nevertheless, several signal amplification techniques have been developed in order to compensate the mentioned weakness, allowing sensitive colorimetric determination of different analytes *via* AuNP-based LFAs. Silver amplification is one of those methods, which is based on nucleation of silver upon gold surface at the test or control lines of LFA strips, enhancing the intensity of the obtained colorimetric signal (Anfossi et al., 2013; Bishop et al., 2019). Silver enhancement technique has been reported in several studies as a simple and efficient method to achieve high sensitivities in LFAs. Rodríguez et al. investigated different approaches to perform silver enhancement in LFAs in order to find out the most optimal one to detect prostatespecific antigen (PSA). They demonstrated that with the silver enhancement a 3-fold sensitivity improvement can be achieved for detection of PSA at levels as low as 0.1 ng/mL (Rodríguez et al., 2016). In another work, silver enhancement was utilized to develop a highly sensitive LFA for detection of R. solanacearum as a dangerous pathogen in agricultural crops and a 10-fold improvement (LOD 200 CFU/mL) in sensitivity was observed (Panferov et al., 2016). In a similar work, a highly-sensitive LFA incorporating silver enhancement was developed for rapid detection of potato leafroll virus (PLRV), where the sensitivity (detection limit 0.2 ng/mL) was improved as a 15-fold compared to conventional LFA (Panferov et al., 2018). The applicability of silver enhancement was also demonstrated for competitive lateral flow assays as well as regular sandwich assays, where the concertation of ochratoxin A (OTA) was measured in beverages with a 10-fold improvement in sensitivity (Ye and Xia, 2018). This work presents development of a paper-based lateral flow assay (LFA) for rapid, low-cost, and user-friendly quantitative determination of IL-6 in the biological samples in a POC manner. This assay uses gold nanoparticles as the colorimetric recognition tags which are conjugated with the specific IL-6 detection antibodies. Since the target detection limit for IL-6 is extremely low (i.e., in pg/mL range), a signal amplification method is incorporated with this colorimetric assay in order to reach the desired sensitivities. Herein, silver amplification technique has been used as a simple, low-cost, and viable amplification approach to enhance the color intensity of the obtained colorimetric signals for IL-6 detection *via* the LFA strips. Despite of the demanding sensitivities for IL-6 detection, silver amplification successfully allowed highly sensitive quantitative measurement of IL-6 in both Phosphate buffer saline (PBS) and serum samples with a limit of detection (LOD) as low as 1 and 5 pg/mL, respectively. The specificity and reproducibility of the assay are investigated by performing the corresponding tests in human serum samples in order to further approve the viability and reliability of the developed assay for facile and sensitive detection of IL-6.

## Experimental Section

### Materials

Human IL-6 monoclonal antibody (MAB206), biotinylated polyclonal antibody (BAF206), and mouse IgG (AF007) antibody, along with recombinant human IL-6 protein (206-IL) were all supplied from R&D Systems (Minneapolis, United States). Other recombinant human cytokines including IFN-*γ*, IL-2, IL-10, and IL-1*β* were also all purchased from R&D Systems and used in the specificity tests. PBS (0.01 M, pH 7.4), streptavidin, sucrose, citric acid, boric acid, Tween-20, Tris, sodium tetraborate, bovine serum albumin (BSA), silver nitrate, sodium azide, hydroquinone, and human serum were purchased from Sigma-Aldrich (Castle Hill, NSW, Australia). Glass fiber sample pad (SB06), absorbent pad, nitrocellulose membrane, and backing pad were purchased from Shanghai Kinbio Tech. Gold nanoparticles (AuNPs, 60 nm) were acquired from Ted Pella, Inc. (Redding, CA, United States). Water was treated with a Millipore (Bedford, MA, United States) Milli-Q water purification system and was used throughout.

### Equipment

A guillotine strip cutter (ZQ 2002), a XYZ dispenser (HM3035), and a vacuum drying oven (PH050A) were all supplied from Kinbio Tech. Co., Ltd. (Shanghai, China) and implemented throughout for fabrication of LFAs. The quantitative image analysis was carried out using a standard LFA reader (Ax-2x, Axxin, Victoria, Australia). Meso Scale Discovery (MSD) human IL-6 kit (Meso Scale Diagnostics, LLC) was supplied from Bio-Strategy Pty Ltd., Australia and used to validate the measured IL-6 concentrations in the tested serum samples.

### Conjugation of Interleukin 6 Antibodies With Gold Nanoparticles

Initially, 1 mL of the colloidal gold solution was taken and its media (i.e., citrate buffer) was replaced by sodium borate buffer (2 mM, pH = 8.5) *via* centrifugation at 7,000 rpm, for 10 min. Then, 5 μL of IL-6 human monoclonal antibody (0.2 mg/mL) was added into the AuNP solution and incubated for 1 h at room temperature (RT) with gentle agitation. Afterward, 1% w/v BSA was added into the conjugate solution as a blocking agent and incubation was carried out for another 30 min at RT. Finally, the solution was centrifuged (7,000 rpm for 10 min) and the supernatant was discarded and the acquired pellets were redispersed in the storage buffer (Tris-HCL 10 mM and pH = 8.2 containing BSA 1% w/v, Tween-20 1% v/v, sucrose 5% w/v, and sodium azide 0.01% w/v).

### Preparation of Lateral Flow Immunoassays Strips

The nitrocellulose membrane (300 × 25 mm) was attached to a plastic backing pad in order to provide the required support for the fragile NC membrane. The supported NC membrane was placed on the dispenser and the corresponding reagents were dispensed upon the T-line and C-line zones. The T-line reagent solution comprised of the biotinylated polyclonal human IL-6 antibody (0.2 mg/mL) and streptavidin (1 mg/mL) at a 1:1 mix ratio, which was preliminary incubated at RT for 1 h. Goat anti-mouse IgG antibody (0.5 mg/mL) was directly dispensed at the C-line zone. Dispensing was performed with defined setting parameters including a dispensing volume of 0.3 μL/cm and speed of 100 mm/s. After dispensing, the NC membrane was dried in a vacuum oven at 37°C for 2 h. Meanwhile, the sample pad (300 × 25 mm) was treated with the treatment buffer (PBS pH = 7.4 containing BSA 1% w/v, Tween-20 0.25% v/v, sucrose 2%) and dried at 37°C for 2 h. Finally, the sample pad and absorbent pad (300 × 25 mm) were attached to the backing pad, overlapping the NC membrane with a 2 mm margin. The complete LFA strip was then cut into individual strips (30 × 5 mm) for further use.

### Lateral Flow Immunoassay Protocol

**SCHEME 1:**
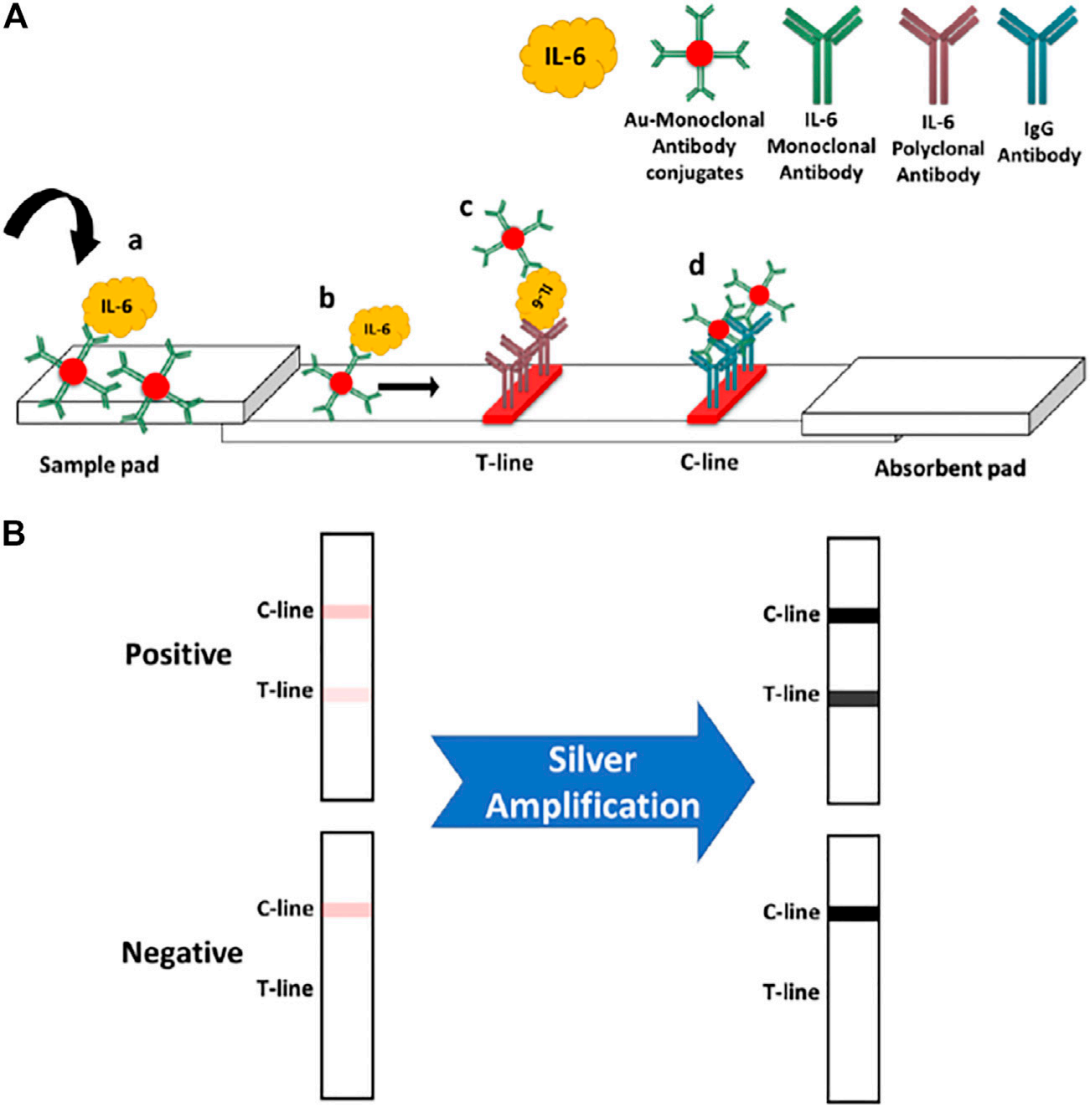
**(A)** standard IL-6 assay principle: 1) IL-6 analyte will bind with the monoclonal antibody conjugated on AuNPs. 2) The IL-6-conjugate complex will move toward the T and C zones. 3) The formed complex will be captured at the T-line by immobilized IL-6 polyclonal antibody. 4) Free excess conjugates will travel further along the strip until they reach the C-line zone and are captured by the anti-IgG antibody. **(B)** Effect of silver amplification on the obtained colorimetric signals upon the paper strips.

IL-6 standard solutions were prepared by diluting a stock solution (100 μg/mL) of IL-6 in sample buffer (PBS with BSA 0.1% w/v). The volume of used Au-antibody conjugate was optimized in order to find out the least amount of required conjugate while generating the highest assay sensitivity. The standard LFA (i.e., no amplification) was performed by adding 5 μL of Au conjugate into 65 μL of IL-6 sample, followed by incubation at RT for 5 min. Afterwards, the sample was loaded into the sample pad of paper strips and then the signal readout was carried out after 15 min using the Axxin reader. Basically, the presented IL-6 LFA is a sandwich immunoassay, implementing AuNPs-antibody conjugates as the colorimetric signalling tags. As shown in Scheme 1A, the IL-6-Au-anti-IL-6 antibody conjugate complex will be loaded into the sample pad of strips which will then move toward the T-line and C-line zones. At the T-line, the complex will be captured by the immobilized polyclonal anti-IL-6 antibody. The excess of conjugates will move even further, where they are captured by the IgG antibody present at the C-line. The appearance of two red lines indicates a positive response. However, if there is no IL-6 analyte present in the introduced sample (i.e., no complex), there will not be any color band formed at the T-line, indicating a negative response.

### Silver Enhancement Procedure

After performing the standard assay (as described above), the silver amplification was applied on the strips in order to enhance the generated colorimetric signals. Before loading the enhancement solution, the absorbent pads of paper strips were dried up by tissue papers (sucking up the accumulated solution) in order to recover their absorption capacity, which was obviously diminished through the standard assay. The silver enhancement solution was prepared freshly by mixing a 1:1 ratio of silver nitrate (0.5% w/v in water) and hydroquinone (3% w/v in 0.5 M citrate buffer, pH = 4.0). 65 μL of the enhancement solution was introduced into the sample pad of strips and then strips were immediately transferred into a dark place for silver enhancement process. Subsequently, the amplified signals were measured after 15 min. In the case of 100% human serum sample, the assay reading time was 30 min. The working principle of silver amplification is shown in Scheme 1B. Herein, the intensity of obtained color bands (colorimetric signals) in the standard assay will be enhanced through the amplification process.

## Results and Discussion

### Optimization of Au-Antibody Conjugate’s Volume

**FIGURE 1:**
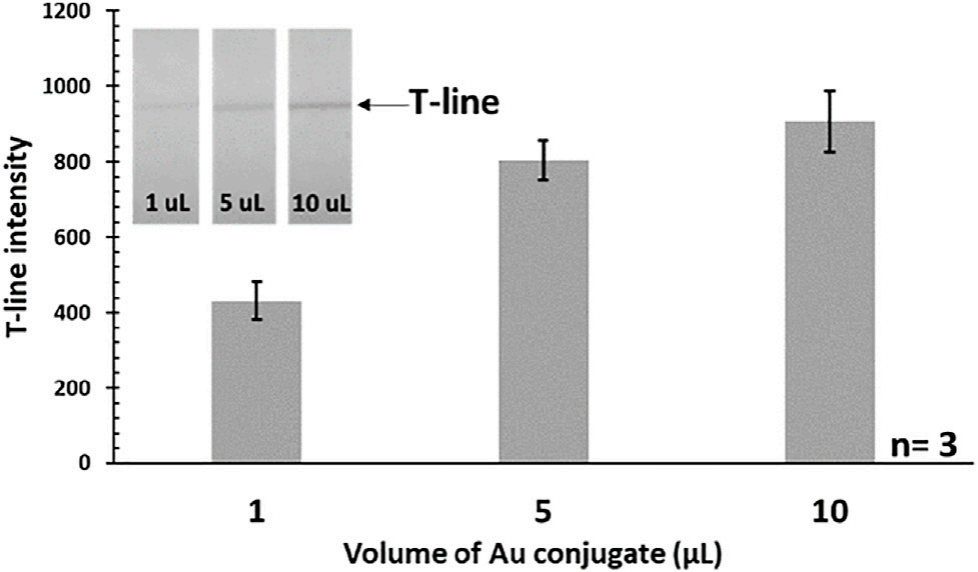
Effect of the used Au-antibody conjugate’s volume on the T-line intensity (IL-6 = 100 pg/mL). Actual photograph of paper strips is shown in the inset.

Initially, the volume of Au conjugate was optimized in order to find out the minimum amount of conjugate required to run the assay, while providing the desired sensitivity. Three different volumes (1, 5, and 10 μL) of conjugates were used to run the assay with constant concentration (i.e., 100 pg/mL) of standard target IL-6 analyte. Based on our studies, a 5 μL volume of conjugate was found to be sufficient for creating a visible T-line. Using higher volumes (e.g., 10 μL) would be redundant as it did not make a significant difference on the obtained signal at the T-line. On the other hand, lower amounts (e.g., 1 μL) do not produce signals with enough intensity. The optimization results are shown in Figure 1, depicting the correlation between the conjugate volume and T-line color intensity (i.e., the target signal). As a result, 5 μL of Au conjugate was used throughout as the optimum amount for the corresponding assays.

### Calibration Curve With and Without Amplification

**FIGURE 2:**
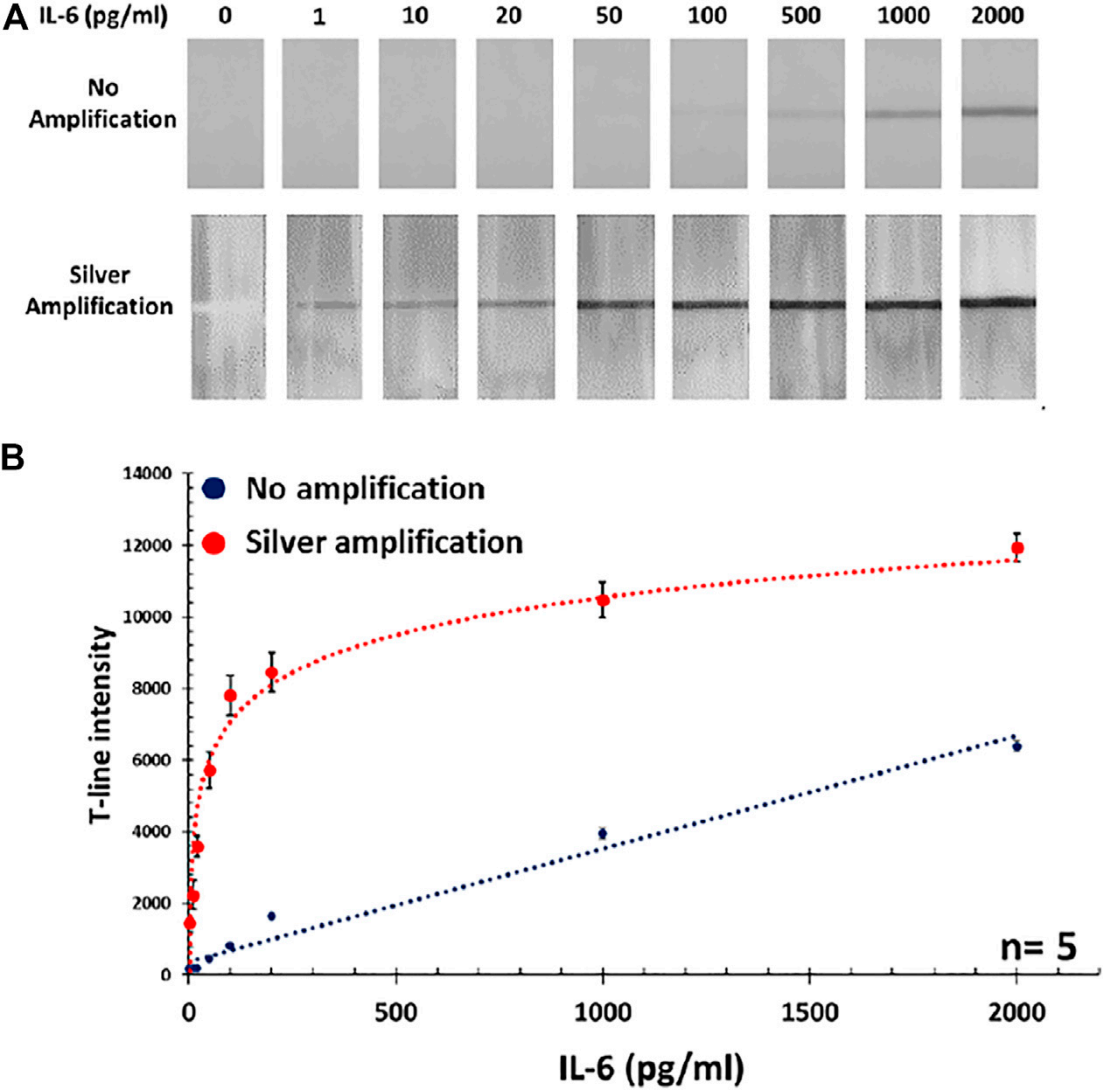
**(A)** Photographs of paper strips showing the intensity of obtained T-lines after and before amplification step. **(B)** The corresponding calibration curve for IL-6 detection in PBS with and without silver amplification.

After finding the optimum volume of Au conjugate, the calibration curve was obtained using standard IL-6 samples (0–2000 pg/mL) in PBS. It was observed that below the concentration of 50 pg/mL, there was no visible color band formed at the T-line, so this was considered as the detection limit of the standard assay. Therefore, the standard assay did not provide the required sensitivity (i.e., 10 pg/mL) and a signal amplification step was necessary in order to reach the target detection limit. Herein, silver amplification technique, as a well-established colorimetric signal amplification method in LFAs, was implemented to resolve this issue. After applying amplification, the intensity of color bands was significantly enhanced and strong signals could be achieved as it was easily visible by the naked eye even at low concentrations below 50 pg/mL. The actual photographs of the paper strips before and after amplification are presented in Figure 2A, comparing the intensity of generated color bands at the T-lines. This resulted in the obtained LOD (i.e., in standard assay) being considerably improved down to 1 pg/mL which was even better than what was required for IL-6 detection. The acquired calibration curve for detection of IL-6 in PBS, with and without amplification is shown in Figure 2B.

### Interleukin 6 Lateral Flow Immunoassay in Human Serum

**FIGURE 3:**
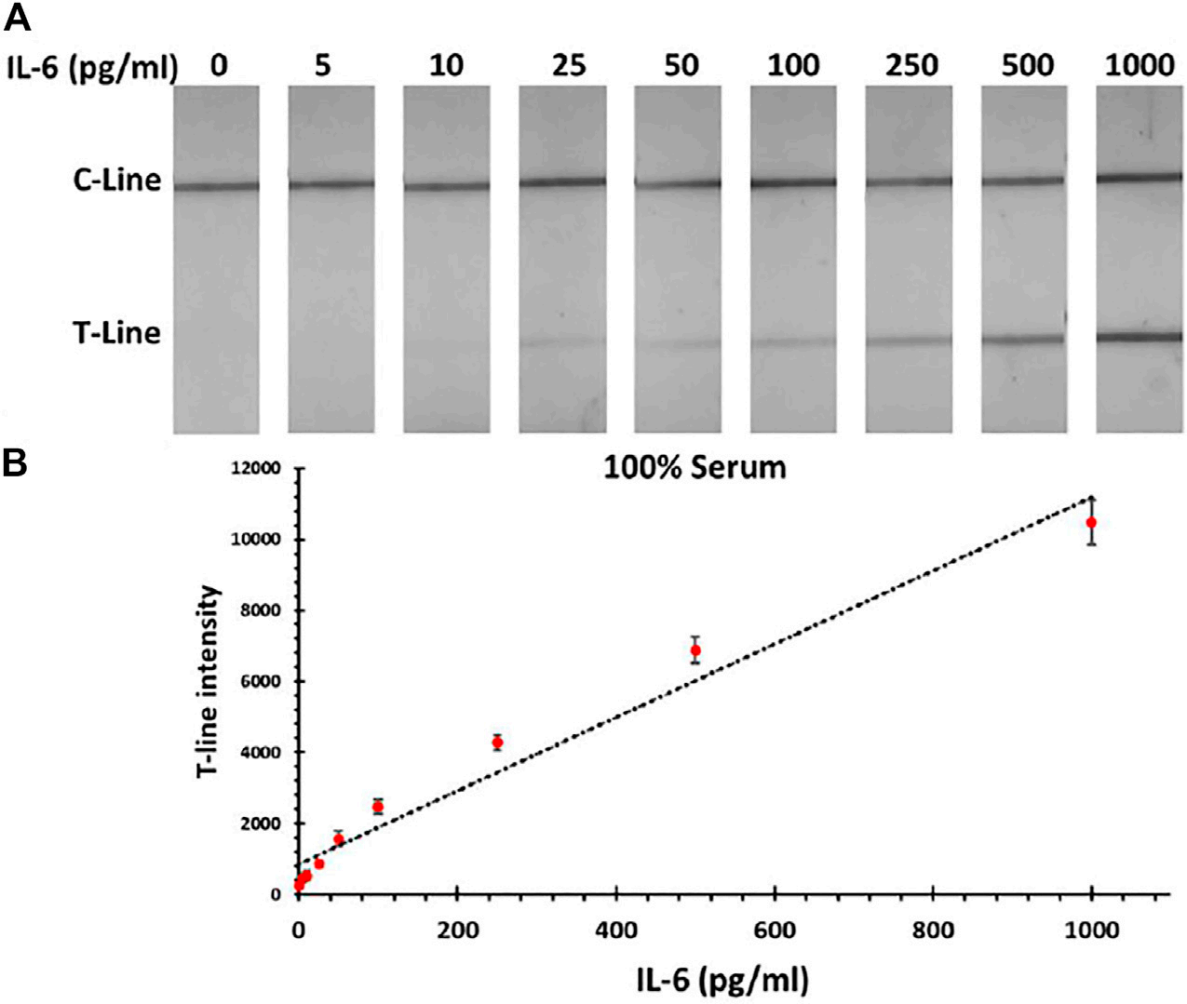
**(A)** Photographs of LFA strips for detection of IL-6 in human serum samples after applying the silver amplification technique. **(B)** Corresponding calibration curve for IL-6 detection in serum sample.

After establishing the IL-6 LFA in PBS, the performance of assay was tested in 100% serum samples spiked with IL-6 protein (5–1,000 pg/mL). As expected, the serum matrix reduced the obtained signal intensity, which consequently impacted the LOD of the standard assay by increasing it up to 200 pg/mL (vs 50 pg/mL for PBS). However, taking advantage of the silver amplification, the IL-6 detection in the required analytical range could be still achieved by having a LOD of 5 pg/mL. These results indicate that silver amplification is a necessity in order to achieve the required sensitivity in the colorimetric IL-6 LFA assay. Photographs of paper strips along with the corresponding calibration curve for IL-6 detection in serum samples are shown in Figure 3. In order to validate the obtained results, MSD system was used in parallel to measure the concentration of IL-6 in serum samples, which illustrated a good agreement with the developed IL-6 LFA (within 10% error).

### Specificity Tests

**FIGURE 4:**
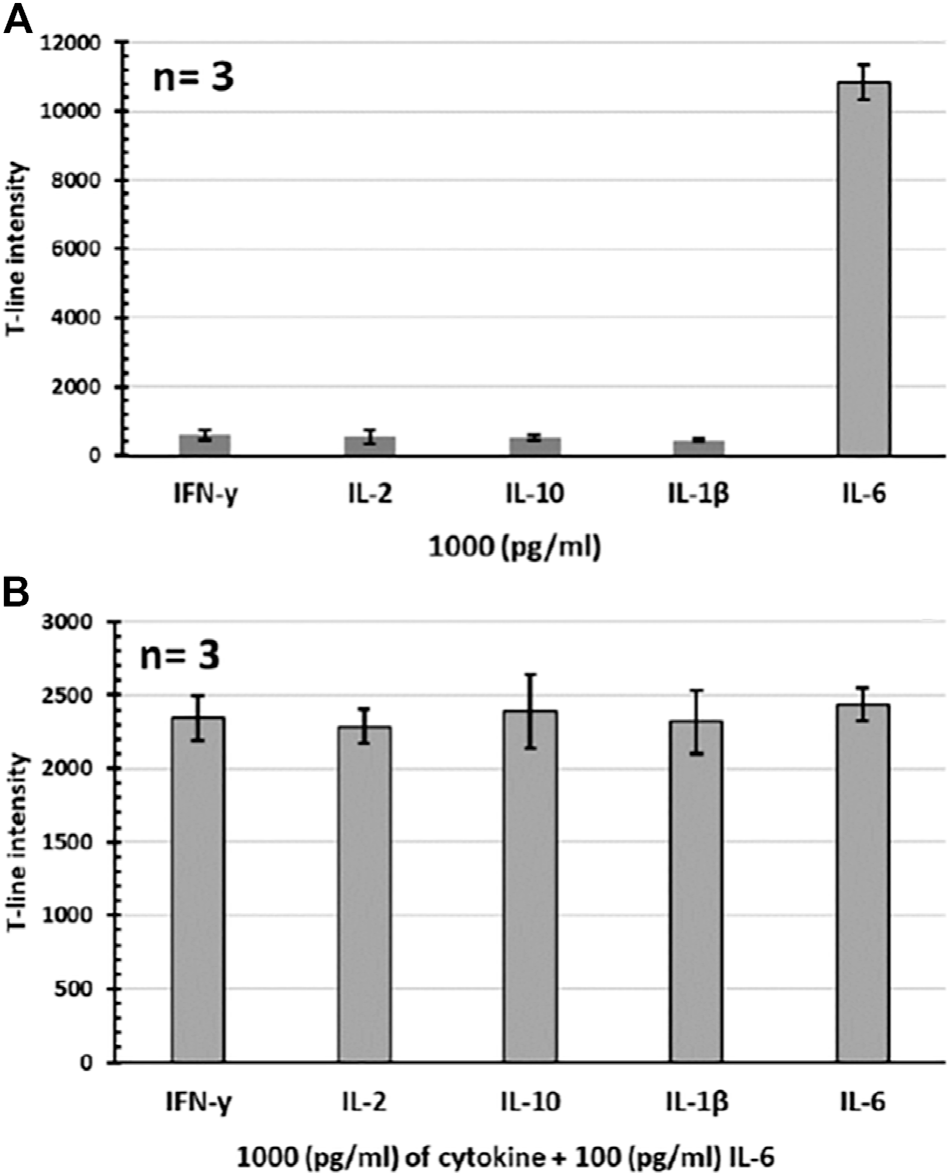
Specificity tests for the developed IL-6 assay. **(A)** Individual cytokine samples. **(B)** IL-6 samples containing excess amount of individual cytokines. All tests were performed in 100% serum sample (*n* = 3).

The specificity test was performed by preparing standard samples (1,000 pg/mL) of different human cytokines (IFN-*γ*, IL-2, IL-10, and IL-1*β*) in serum and then running the assay for each individual sample. It was observed the assay only responds to the target IL-6 sample, while there was no significant signal obtained for other tested cytokine samples, confirming the high specificity of the developed assay. Results regarding these specificity tests are shown in Figure 4A. The specificity test was also further evaluated by running the assay for IL-6 samples (100 pg/mL) containing excess amount (1,000 pg/mL) of other individual cytokines as potential interferences. Results showed that the presence of other cytokines (even at a level of ten times higher than the target IL-6 analyte) does not affect the performance of the assay as no interference was recorded. In other words, the assay is totally selective for the target IL-6 analyte. The assay response in these specificity tests is presented in Figure 4B, showing the almost similar signal intensities (CV% = 7.2) obtained for all tested samples regardless of the type of interfering cytokine present.

### Reproducibility Tests

The reproducibility tests for the developed IL-6 LFAs were carried out by assessing the performance of the test strips in three separate time intervals (1 day, 1 week, 1 month, *n* = 3 for each) after preparation. Herein, two different concentrations (5 and 100 pg/mL) of IL-6 were tested. These results indicate that the assay provides a reasonable reproducibility for colorimetric POC detection of IL-6 analyte. The acquired signals in this regard are presented in Table 1. The batch-to-batch reproducibility was also assessed by preparing three individual batches of IL-6 LFA strips and running the assay for two different concentrations (5 and 100 pg/mL, *n* = 3 for each batch) of IL-6 samples. Similarly, results showed relatively consistent signal intensities obtained from different batches of test strips. The average measured signals for each tested concentration along with the calculated CV% are presented in Table 1.

**TABLE 1:** Reproducibility test. LFA in three different time intervals (1 day, 1 week, and 1 month) and batch to batch test.

IL-6 pg/mL	Batch to batch test		Time intervals	
	3 batches, *n* = 3 at each		3 intervals, *n* = 3 at each	
—	Mean ± SD (T intensity)	CV%	Mean ± SD (T intensity)	CV%
5	594 ± 75	13.4	450 ± 86	19.2
100	2800 ± 101	3.6	2380 ± 304	9.8

## Conclusion

In this work, a paper-based LFA was successfully developed for colorimetric point-of-care quantitative determination of IL-6 in biological samples. This assay incorporates silver amplification technique as a viable, low-cost, user-friendly, and efficient method for enhancing the obtained colorimetric signals from LFAs. The present assay allows detection of IL-6 in serum samples in the dynamic range of 5–1,000 pg/mL with a LOD of 5 pg/mL. The specificity tests indicated the viability of the assay for detection of IL-6 target analyte in real biological samples. The reproducibility tests also represent the reliability of the developed assay for detection of IL-6 in varying conditions. The fully colorimetric nature of the developed assay presents potentials for applicability of this assay in point-of-care detection of such a significant biomarker (i.e., IL-6) in low-resource settings. Future work remains to develop this system further for detection of IL-6 in whole blood samples by optimizing both the chemistry and design of the present assay. For instance, a sample preparation comportment (e.g., a plasma separation membrane) can be incorporated into the present LFA strips to filter out the red blood cells, enhancing the visibility of the colorimetric signals. Meanwhile, sensitivity of LFA can be further improved to meet the requirement of clinical detection by applying signal amplification technologies in POCT, such as CRISPR/Cas biosensing system (Liu et al., 2019; Dai et al., 2020; Lin et al., 2021; Shirshahi and Liu, 2021). Furthermore, the colorimetric feature of this assay allows further developments targeting smartphone-based POC detection of IL-6 and also other similar cytokines. Therefore, a smartphone-based readout system (i.e., a smartphone holder/light box) can be coupled with this assay to replace the herein implemented expensive LFA reader, enabling on-site, facile, and rapid image analysis for IL-6 detection. Finally, this work can be extended for detection of other human cytokines such as IFN-*γ*, IL-2, IL-10, IL-1*β*, et al.

## Data Availability

The original contributions presented in the study are included in the article/Supplementary Material, further inquiries can be directed to the corresponding authors.

## Abbreviations

CV, coefficient variance; SD, standard deviation
